# Ammonia Emissions from NPK Fertilizer Production Plants: Emission Characteristics and Emission Factor Estimation

**DOI:** 10.3390/ijerph19116703

**Published:** 2022-05-31

**Authors:** Seongmin Kang, Goeun Kim, Joonyoung Roh, Eui-chan Jeon

**Affiliations:** 1 Climate Change & Environment Research Center, Sejong University, Seoul 05006, Korea; smkang9804@gmail.com; 2 Department of Climate and Energy, Sejong University, Seoul 05006, Korea; go1130@hanmail.net; 3 Department of Climate and Environment, Sejong University, Seoul 05006, Korea; jy.roh0925@gmail.com

**Keywords:** ammonia emission, fertilizer production plant, emission factor

## Abstract

Fertilizers are made from manure, but they are also produced through chemical processes. Fertilizer is an ammonia emission source; it releases ammonia when used. Ammonia is also emitted during the production process. Although many studies related to fertilizer application have been conducted, there are few research cases related to the production process and related emissions are not calculated. In this study, the ammonia emissions from NPK (nitrogen phosphorus Potassium oxide) fertilizer production facilities were checked through actual measurement and related characteristics were analyzed. In addition, emission factors were developed, and the necessity of developing emission factors was also confirmed. As a result of the development of the emission factor, it was found to be 0.001 kgNH_3_/ton, which is like the range of emission factors in related fields. The NPK ammonia emission factor of this study was found to be higher than the minimum emission factor currently applied in South Korea, and it was judged to be a level that can be used as an emission factor.

## 1. Introduction

The concentration of PM_2.5_ in South Korea was 19 μg/m^3^ in 2020, approximately 21% lower than that in the previous year, owing to the COVID-19 pandemic and the implementation of policies on particulate matter [1]. However, the concentration is considerably higher than the strict annual average limit of 5 μg/m^3^ implemented by the World Health Organization (WHO) in 2021. Additionally, the concentration of PM_2.5_ in 55 cities in South Korea exceeds the national annual average limit of 15 μg/m^3^, indicating that the management of particulate matter requires considerable attention [2].

Secondary products account for approximately 72% of fine particulate matter emissions in South Korea. Sources of secondary particulate matter include NH_3_, NOx, SOx, and volatile organic compounds [3,4]. While South Korea is striving to reduce air pollutants in various ways, current policies are mainly focused on the management of NOx and SOx. Little is known about the emission sources and application of appropriate emission factors of NH_3_ in South Korea [5,6]. Therefore, relevant studies are required to ensure the reliability of inventories.

NH_3_ emissions in South Korea were 315,975 tons in 2018. The agricultural sector accounted for the largest proportion (79%) of NH_3_ emissions, with manure management and cropland sectors as major sources [7,8,9]. NH_3_ emissions during the application of fertilizers in the cropland sector have been evaluated in several studies. However, NH_3_ emissions from fertilizer production facilities have not been monitored and only phosphate fertilizers are included in the calculation of PM_2.5_ emissions during fertilizer production [10]. Therefore, it is necessary to quantify NH_3_ emissions and calculate and establish an accurate emission factor.

In this study, NH_3_ emissions were measured at NPK fertilizer production facilities and monthly concentrations and seasonal effects were evaluated in a statistical framework. NH_3_ emission factors were determined using the measured data and the need to develop a national emission factor was confirmed by comparing newly calculated emission factors with reported values in analogous fields.

## 2. Methods

### 2.1. Selection of Facilities

Samples were collected from four NPK fertilizer production facilities to confirm the NH_3_ concentration and emission characteristics. Facilities that annually produced more than 50,000 tons of NPK fertilizers were selected as targets. Table 1 shows the average annual production amount at the target facilities and the number of samples. Samples were collected for 3 to 4 years. It was attempted to measure the monthly concentration data per facility but since the maintenance period for each facility is different and the annual maintenance period is not the same, more than 25 samples were collected for each facility.

### 2.2. NH_3_ Analysis at NPK Fertilizer Production Plants

The indophenol method was used to measure NH_3_ emissions at the NPK fertilizer production facilities following the standard odor test method and the standard air test method specific to the measurement of NH_3_ in South Korea [11,12]. In the indophenol method, NH_3_ is quantified by adding a phenol-sodium nitroprusside solution and a sodium hypochlorite solution to the sample and measuring the absorbance of indophenols produced in the reaction with ammonium ions.

**Figure 1:**
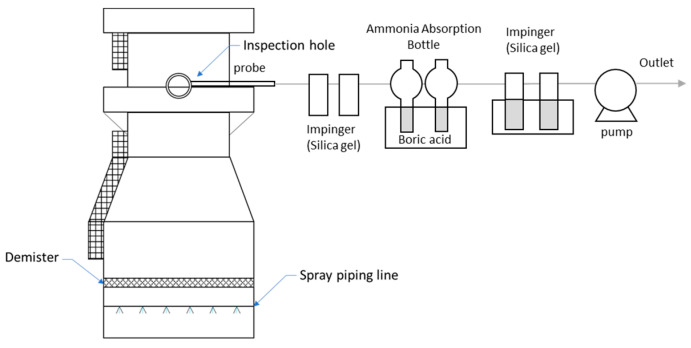
Schematic of the field setup for ammonia sampling at NPK fertilizer production plants.

To sample NH_3_, 25 mL of boric acid was added as an NH_3_ absorbent in two 50 mL flasks each, then 50 L of exhaust gas was pulled in by a mini-pump (SIBATA MP-ΣNII, Tokyo, Japan) at 4 L/min for about 13 min. Figure 1 shows a schematic diagram of the NH_3_ sampling technique. The absorbance of the absorbent solution containing NH_3_ was measured at a wavelength of 650 nm using a spectrophotometer (Shimadzu 17A, Kyoto, Japan). NH_3_ was sampled through an inspection hole.

### 2.3. Development of the NH_3_ Emission Factor at NPK Fertilizer Production Plants

The emission factor of gaseous pollutants (e.g., air pollutants) indicates the number of emissions in a sample and is calculated based on the flow rate and the concentration of the emitted pollutant, accounting for combustion and production [10]. In this study, the equation used in previous studies was adopted to determine the NH_3_ emission factor for the NPK fertilizer production facilities, as shown in Equation (1) [13,14]. The flow rate and NPK fertilizer production amount necessary to develop the NH_3_ emission factor for the NPK fertilizer production facilities were obtained from the data provided by the target facilities, and the daily mass flow rate was used.

(1) $$EF_{NH_{3}}=\left[C_{NH_{3}}\times \frac{M_{w}}{V_{m}}\times Q_{day}\times 10^{-6}\right]/FC_{day}$$

where EF is the emission factor (kg NH_3_/ton), $C_{NH_{3}}$ is the NH_3_ concentration in exhaust gas (ppm), $M_{w}$ is the molecular weight of NH_3_ (constant) = 17.031 (g/mol), $V_{m}$ is one mole ideal gas volume in standardized condition (constant) = 22.4 (10^−3^ m^3^/mol), $Q_{day}$ is the daily accumulated flow rate (Sm^3^/day) (based on dry combustion gas), and $FC_{day}$ is the daily NPK production (ton/day).

### 2.4. Uncertainty Analysis of the NH_3_ Emission Factor Using a Monte Carlo Simulation

**Figure 2:**
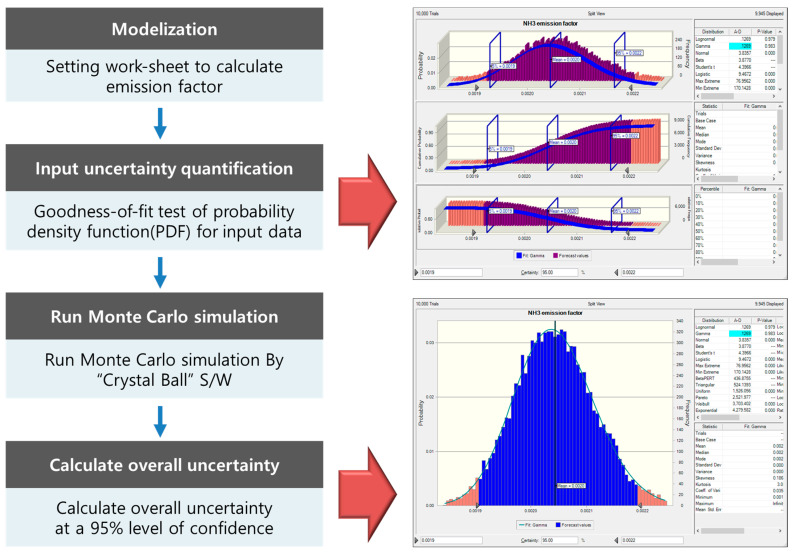
Process of the Monte Carlo simulation for estimating the uncertainty of the emission factor.

In South Korea, the uncertainty of air pollutant emission estimates was identified in accordance with the EPA Data Attribute Rating System (DARS) methodology, which is based on expert input [15,16]. However, the European EMEP/EEA suggests that quantitative uncertainty assessments of emission factors should be conducted in accordance with the methodology of IPCC 2006 in greenhouse gas inventories [17]. In this study, the Monte Carlo simulation proposed by IPCC 2006 was used to quantitatively evaluate the uncertainty of the NH_3_ emission factor [18]. Widely used in environmental fields, Monte Carlo simulations can be used to evaluate uncertainty by random sampling and specifying a probability density function (PDF) for input variables [19,20]. The Monte Carlo simulation consists of four stages, as summarized in Figure 2. In the first step, a model is selected. For this, a worksheet is constructed to calculate the greenhouse gas emission factor. Second, the PDF of the input variables is validated. In this study, the significance level for testing the hypothesis was set at 5%. Then, the PDF is calculated for each variable through fitness analysis of the data required for NH_3_ emission factor estimation, such as the NH_3_ emission concentration, emission flow rate, and fertilizer production amount. Third, the Monte Carlo simulation is performed using the Crystal Ball program for simulations with random sampling. Fourth, the uncertainty range at the 95% confidence interval is calculated based on the simulation results.

## 3. Results and Discussion

### 3.1. Characteristics of NH_3_ Emissions at NPK Fertilizer Production Plants

Table 2 shows the NH_3_ concentrations at the NPK fertilizer production facilities (referred to as facilities A–D). The NH_3_ concentration at the NPK fertilizer production facilities ranged from 0.01 ppm to 1.48 ppm.

The concentration at facility A ranged from a minimum of 0.01 ppm to a maximum of 1.48 ppm, with an average value of 0.32 ppm and a standard deviation of 0.33 ppm. The concentration at facility B ranged from 0.01 ppm to 0.82 ppm, with an average concentration of 0.18 ppm and a standard deviation of 0.33 ppm. The concentration at facility C ranged from 0.06 ppm to 1.14 ppm, with an average of 0.43 ppm and a standard deviation of 0.28 ppm. The concentration at facility D ranged from 0.01 ppm to 1.31 ppm, with an average of 0.35 ppm and a standard deviation of 0.27 ppm.

**Figure 3:**
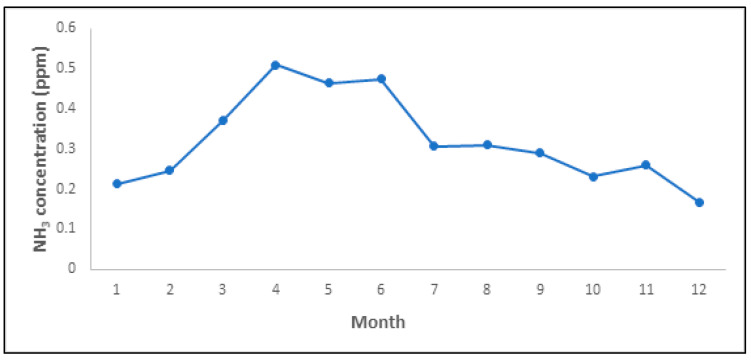
Monthly trend of NH_3_ concentrations at NPK fertilizer production plants.

To understand the temporal characteristics of NH_3_ emissions at the NPK fertilizer production facilities, trends in the monthly average concentrations of NH_3_ were examined (Figure 3). In the case of NPK fertilizer manufacturing facilities, depending on the facility, the maintenance period may be as low as 1 month to as high as 3 months. In this study, in order to confirm the monthly NH_3_ emission concentration of the overall NPK fertilizer manufacturing facilities, the monthly average data of the four facilities were used to confirm the temporal emission characteristics. The concentration was the highest from March to June. These high concentrations could be attributed to the high activity during this period, if the NPK fertilizer production amount at the target facilities (8000–9000 tons/month) exceeded the monthly average of 6926 tons/month.

Furthermore, the seasonal differences in NH_3_ emitted from the NPK fertilizer production facilities were analyzed. Table 3 shows the NH_3_ concentration data for different seasons. The average concentration was the highest in the spring (0.45 ppm), followed by the summer, fall, and winter. These results are consistent with those of the analysis of monthly average concentrations, revealing that NH_3_ emissions were high in the spring months of March to May and in the summer month of June.

For statistical analyses, SPSS 21 (IBM, New York, NY, USA) was used to verify the normality of the NH_3_ concentration data (to determine whether parametric or nonparametric statistical tests should be applied) and to analyze differences among seasons.

In general, the Kolmogorov–Smirnov (K–S) and Shapiro–Wilk tests are widely used to test normality, depending on the number of samples. The K–S test is typically used when the number of samples is greater than 2000, whereas the Shapiro–Wilk test is used when there are fewer than 2000 samples.

In these tests, the null hypothesis is that the data are normally distributed and this hypothesis is rejected when *p* < 0.05. To confirm the seasonal effect, mean values can be compared using one-way ANOVA (for normally distributed data) and Kruskal–Wallis tests (for non-normal distribution).

In this study, concentration data obtained at the NPK fertilizer production facilities were evaluated for normality, as shown in Table 4. Since the sample size of the NH_3_ concentration estimates was less than 2000, normality was tested using the Shapiro–Wilk method, revealing a non-normal distribution. Accordingly, the Kruskal–Wallis test was used to analyze seasonal differences.

The seasonal differences in the NH_3_ concentration of the NPK fertilizer production facilities were analyzed using the Kruskal–Wallis test and the results are summarized in Table 5. Based on the Kruskal–Wallis test, the seasonal differences were significant (*p* < 0.05).

When the null hypothesis is rejected, a post hoc test must be conducted. The post hoc test, used to identify the cause of the seasonal differences, can be performed by reviewing the seasonal response data. The results of this analysis are provided in Table 6. The difference in emissions between the spring and winter was significant (*p* < 0.05), indicating seasonal differences in the overall result. While most seasonal differences were not significant, the difference in concentrations between the spring and winter was significant, which should be considered when developing the corresponding emission factor.

### 3.2. NH_3_ Emission Factor and NH_3_ Emissions at NPK Fertilizer Production Plants

In this study, 139 NH_3_ samples were collected from four NPK fertilizer production facilities to calculate the NH_3_ emission factor (Table 7). The overall NH_3_ emission factor for the NPK fertilizer production facilities was 0.001 kg NH_3_/ton. The NH_3_ emission factor for most facilities was 0.001 kg NH_3_/ton; however, that of facility C was 0.002 kg NH_3_/ton. This is consistent with the observation that facility C had the highest NH_3_ concentration.

Although the emission factor of the NPK fertilizer production facilities should be compared to emission factors in other regions and countries, related studies are lacking. Therefore, the emission factors in analogous fields were used for comparison, confirming the necessity to develop a national NH_3_ emission factor.

Additionally, since this study was mainly focused on large-scale sites that were well-equipped with elutriation-type odor prevention facilities, such as scrubbers, absorption towers, and wet scrubbers, the concentrations of NH_3_ were low. We attempted to measure NH_3_ concentrations prior to the implementation of preventive measures; however, receiving cooperation from companies was difficult owing to safety issues. Therefore, the NH_3_ emission factor was estimated at sites with no prevention facilities to investigate the prevention efficiency and an uncontrolled emission factor was proposed by calculating an additional emission factor based on the concentration without the application of preventive measures. Regarding the treatment efficiency of the prevention facilities, we were able to obtain relevant data through an interview with the person in charge of the relevant facility.

Analysis of the effectiveness of the prevention facilities revealed that all target companies exhibited a treatment efficiency of 90% or higher. Therefore, a treatment efficiency of 90% was used for the prevention facilities for the calculation of the emission factor, as shown in Equation (2).

(2) $$C_{INPUT}=\frac{C_{OUPUT}}{1-E_{P}}$$

where $C_{INPUT}$ is the NH_3_ concentration at the front of the prevention facilities (ppm), $C_{OUPUT}$ is the NH_3_ concentration at the back of the prevention facilities output (ppm) and $E_{p}$ is the effectiveness of the prevention facilities.

Table 8 shows a comparison between foreign NH_3_ emission factors reported in analogous fields, uncontrolled NH_3_ emission factors without considering the efficiency of prevention facilities, and NH_3_ emission factors calculated at the final emission outlet.

Since no reports of NH_3_ emission factors at NPK fertilizer production facilities were found, emission factors for the NH_3_ production sector in the same chemical industry were used for comparison. In the comparative analysis, the NH_3_ emission factor at the final outlet following treatment by the prevention facilities demonstrated a 50-fold difference. The NH_3_ emission factor in the absence of prevention facilities showed a 5-fold difference. However, the NH_3_ emission factor in this study was comparable to the lower limit of the NH_3_ emission factor of the NH_3_ production facilities at the 95% confidence level, suggesting that our results were valid. Furthermore, the uncontrolled NH_3_ emission factor fell between the upper and lower limits of the foreign emission factor and was valid.

Among the NH_3_ emission factors currently applied in South Korea, the factor used for bituminous coal is the smallest (0.00028 kgNH_3_/ton), approximately 3.5 times higher than the values obtained in this study. Therefore, it is necessary to develop and apply an NH_3_ emission factor from the perspective of inventory.

### 3.3. Uncertainty in the NH_3_ Emission Factor at NPK Fertilizer Production Plants

**Figure 4:**
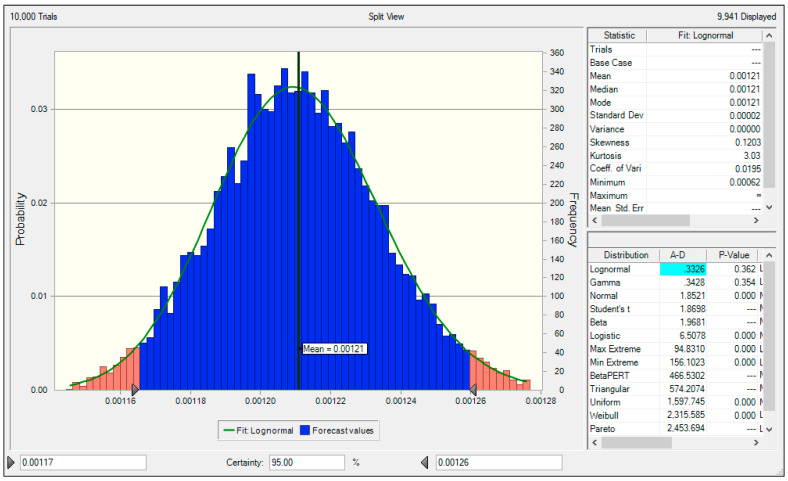
Uncertainty of NPK fertilizer production facilities NH_3_ emission factor.

A Monte Carlo simulation was used to evaluate the uncertainty of the NH_3_ emission factor for the NPK fertilizer production facilities calculated in this study (Figure 4). The Monte Carlo simulation was implemented in Crystal Ball.

The PDF of the NH_3_ emission factor for the NPK fertilizer production facilities calculated in this study had a lognormal distribution. The mean value was 0.00121 kgNH_3_/ton and the lower 2.5% and upper 97.5% quantiles at the 95% confidence level were 0.00117 tonNH_3_/m^3^ and 0.00126 tonNH_3_/m^3^, respectively. Based on these values, the uncertainty of the calculated NH_3_ emission factor at the 95% confidence level was −3.3% to +4.4%.

The lack of reported point estimates or ranges for the NH_3_ uncertainty makes comparative analyses difficult. Insufficient NH_3_ emission factor values for fertilizer production facilities are available for comparison. However, according to the EMEP/EEA inventory guidebook, when the uncertainty of an emission factor is in the range of 10% to 30%, the emission factor is classified as Grade A. The NH_3_ emission factors in this study were classified as Grades D–E, indicating that the uncertainty of the emission factors in this study was high [21].

In the case of South Korea, the uncertainty of air pollutant emission estimates is evaluated in accordance with the DARS method proposed by the EPA of the United States [16]. In the DARS method, scores are assigned to reflect the characteristics of the inventory; however, this often depends on expert opinions and may be influenced by subjectivity. According to the European EMEP/EEA Air Pollution Inventory Guidebook, the uncertainty of pollutant emissions needs to be quantitatively assessed, in a similar manner to that used for greenhouse gases. In particular, the IPCC guideline for calculating greenhouse gas emissions involves Monte Carlo simulations (Approach 2), like the method used to evaluate uncertainty in this study. The IPCC recommends that air pollutant emissions are also reported as indirect greenhouse gases when reporting greenhouse gas emissions [22]. Therefore, it is necessary to develop an uncertainty range for the consistent evaluation of air pollutants and greenhouse gases.

## 4. Conclusions

In this study, NH_3_ was confirmed to be an important omitted source of emissions, as determined by the calculation of NH_3_ emission factors based on field measurement. Our findings reveal the importance of developing a national NH_3_ emission factor in South Korea.

Field measurements were performed for 3 years at four NPK fertilizer production facilities and these data were used to calculate NH_3_ emission factors. The concentration of NH_3_ emitted from the NPK fertilizer production facilities was 0.01–1.48 ppm. To understand the temporal characteristics of NH_3_ emissions at the NPK fertilizer production facilities, the trends in monthly average NH_3_ concentrations were examined. The concentration was the highest from March to June, overall. This result was cross-checked against other relevant data, which indicated that the facilities were running the most actively during this period, with the production of NPK fertilizers (8000–9000 tons/month) higher than the average monthly fertilizer production.

In this study, seasonal differences and causes of variation were characterized. The analysis confirmed significant seasonal differences; in particular, emissions in the spring and winter were significantly different. This was consistent with the analysis results of monthly concentrations and could be attributed to the relatively active fertilizer production during the spring and relatively low production during the winter, resulting in a significant difference. Therefore, data for all seasons should be acquired to ensure the reliability of emissions factors.

The newly calculated NH_3_ emission factor was comparable to the lower limit of the emission factors in analogous fields overseas but was greater than the smallest NH_3_ emission factor applied in South Korea, confirming the need for the development of an NH3 emission factor at the national level.

Analyses of quantitative uncertainty are important when developing emission factors. In this study, Monte Carlo simulations were used to calculate quantitative uncertainty, as suggested in the IPCC guidelines. The PDF for the NPK fertilizer production facilities exhibited a lognormal distribution and the uncertainty range was −3.3% to +4.4% at a 95% confidence level, equivalent to Grade A according to the European EMEP/EEA uncertainty evaluation system. Therefore, the emission factor calculated in this study was reliable to some degree.

The key findings and implications of the study are as follows.

1. The NH_3_ emissions from NPK fertilizer production facilities that do not monitor NH_3_ were confirmed and quantitatively evaluated and the corresponding NH_3_ emission factor was calculated.
2. Based on data measured over 3 years, monthly NH_3_ concentration trends and seasonal characteristics at the NPK fertilizer production facilities were examined and the cause of the seasonal differences was statistically analyzed.
3. The need for a national emission factor was confirmed by comparing the NH_3_ emission factor for the NPK fertilizer production facilities calculated in this study with emission factors reported in analogous cases and those applied in South Korea.
4. The recent Paris Agreement requires more countries to report greenhouse gas emissions. The relevant reporting system also recommends reporting air pollutants as indirect greenhouse gases. In Europe, the reliability of emission factors and emission amounts should be in compliance with air pollutant inventory guidelines. In South Korea, the uncertainty of emission factors and emission amounts are evaluated using an expert input-based ranking evaluation method suggested by the U.S. EPA. However, a quantitative uncertainty assessment is required to ensure the same level of reliability applied to greenhouse gas inventories, as recommended by the European EMEP/EEA.

In this study, uncertainty in the NH_3_ emission factor was evaluated using Monte Carlo simulations, the evaluation method used for greenhouse gases. The quantitative uncertainty estimates provide reference values for other researchers.

Since this study focused on the need to address omitted emission sources by analyzing the NH_3_ emission characteristics and emission factor, this study was limited to a small number of facilities and the results do not reflect emissions from small-scale facilities without preventive measures. The future development of NH_3_ emission factors based on a larger number of facilities will improve the reliability of NH_3_ inventories in the fertilizer production facility sector and, furthermore, will contribute to the establishment of policies for the reduction of PM_2.5_.

## Figures and Tables

**Table 1:** Characteristics of the investigated NPK fertilizer production plants.

Site	Production (Ton/Year)	Sampling
A fertilizer production plant	50,900	44
B fertilizer production plant	59,015	37
C fertilizer production plant	91,009	26
D fertilizer production plant	54,531	32

**Table 2:** NH_3_ concentration of the investigated NPK fertilizer production plants.

Site	Mean (ppm)	Min (ppm)	Max (ppm)	SD (ppm)	Sampling
A fertilizer production plant	0.32	0.01	1.48	0.33	44
B fertilizer production plant	0.18	0.01	0.82	0.18	37
C fertilizer production plant	0.43	0.06	1.14	0.28	26
D fertilizer production plant	0.35	0.01	1.31	0.27	32

**Table 3:** Seasonal characteristics of NH_3_ concentrations at NPK fertilizer production plants. Unit: ppm.

Season	Mean	Std. Deviation	Minimum	Maximum	Sampling
Spring	0.45	0.41	0.02	1.83	35
Summer	0.37	0.31	0.01	1.26	35
autumn	0.26	0.25	0.02	0.91	36
Winter	0.24	0.24	0.001	0.91	33

**Table 4:** The result of the normality test of NH_3_ concentrations at NPK fertilizer production plants.

Normality Test Result		Shapiro-Wilk		
		Statistic	Degree of Freedom, Df	Sig.
NPK fertilizer production plants	Spring	0.279	34	<0.0001
	Summer	0.144	35	0.003
	Autumn	0.212	37	<0.0001
	Winter	0.179	47	<0.0001

**Table 5:** The result of the Kruskal–Wallis test by NH_3_ concentration at NPK fertilizer production plants.

Hypothesis Test	Null Hypothesis	Test	Sig.	Decision
NPK fertilizer production plants	The distribution of NH_3_ is the same across categories of season	Independent Samples Kruskal–Wallis Test	0.008	Reject the null hypothesis

**Table 6:** The result of the pairwise comparison test by NH_3_ concentration at NPK fertilizer production plants.

Pairwise Comparisons of Season	Adjusted Significant *	Test Statistic
Winter-Autumn	1.00	3.169
Winter-Summer	0.201	21.023
Winter-Spring	**0.016**	29.915
Autumn-Summer	0.524	17.854
Autumn-Spring	0.066	26.745
Summer-Spring	1.00	8.891

* The result of Bonferroni correction.

**Table 7:** NH_3_ emission factors of the investigated NPK fertilizer production plants.

Site	Mean (kg NH_3_/ton)	SD (kg NH_3_/ton)	Sampling
A fertilizer production plant	0.001	0.001	44
B fertilizer production plant	0.001	0.002	37
C fertilizer production plant	0.002	0.001	26
D fertilizer production plant	0.001	0.001	32
Total	0.001	0.001	139

**Table 8:** Comparison of NH_3_ emission factor in similar fields.

This Study		EEA (2019)		
NPK fertilizer production plants		Ammonia production		
NH_3_ emission factor at stack (kgNH_3_/ton)	Uncontrolled NH_3_ emission factor (kgNH_3_/ton)	NH_3_ emission factor (kgNH_3_/ton)	95% confidence interval	
			Lower	Upper
0.001	0.011	0.05	0.001	0.1

## Data Availability

Data sharing not applicable.
